# Involving peers in research: the UNSW community reference panel

**DOI:** 10.1186/s12954-019-0325-3

**Published:** 2019-08-30

**Authors:** Melinda Walker, Kim Beadman, Steve Griffin, Mitchell Beadman, Carla Treloar

**Affiliations:** Centre for Social Research in Health, UNSW Sydney, Sydney, Australia

## Abstract

There is limited literature about how to best “do” community involvement in research and no one model of community involvement in research that has been shown to be more effective than others. This paper presents one way to receive the input of people with experiences relevant to research with marginalised groups, including people who use and inject drugs. The UNSW Community Reference Panel is a virtual network of people from across Australia who are engaged to provide input and consultation on research design, processes, materials, and outputs. Although this panel goes some way towards community involvement and consultation in the research process, it does not take the place of other aspects of community governance and ownership, especially as informed by principles of research with Indigenous peoples. This model is an example of a means to bring the voices and perspectives of people who are generally excluded from the research and decision-making structures that affect their lives, including people who inject drugs, to influence the questions that are asked in research, how research gets done, and to what purpose research findings are put.

There are numerous reasons why people affected by research should be involved in the research. These reasons could be considered as instrumental (to improve the quality of research and community understanding of research) and as ethical practice (the right of people affected by research to participate in the decision-making process). The literature in this area outlines the ways in which community involvement can support researchers to do better in all aspects such as posing a research question that better reflects community need, recruitment processes that are more effective, and ethical and interpretation of findings which better reflect the community’s experiences and expectations. The Australian National Health and Medical Research Council (NHMRC) recognises both the instrumental and ethical arguments in its recognition that “involving consumers and community members can add value to health and medical research and have a right and responsibility to do so” [1] (p. 8).

The nature of peer involvement in research as an ethical practice is highlighted in discussions of people who inject drugs. A key guiding document was published by the Canadian HIV/AIDS Legal Network in 2005 with the emblematic title of “Nothing about us without us” [2] which was informed by a 2002 national statement on ethical issues in research with people who inject by the Australian drug user organisation, AIVL [3]. This landmark document notes that, in practice, “people who use drugs should be invited to participate in all consultations, committees, or fora where policies, interventions, or services concerning them are planned, discussed, researched, determined, or evaluated” (p. 7).

There is no one model of community involvement in research that has been shown to be more effective than others [4]. However, most models assume an ongoing involvement of individuals across the lifetime of a research project or a long term relationship between researchers and community [5]. While these are undoubtedly important models of genuine community involvement, these arrangements may not be achievable or appropriate in all situations [6], can cause some frustration for community members related to lengthy processes, transportation and attendance requirements [4], and may further exclude people already experiencing marginalisation or inequalities [7]. At UNSW, we developed a different model of engagement to meet the needs of numerous research projects.

The UNSW Community Reference Panel is a virtual network of people from across Australia who are engaged to provide input and consultation on research design, processes, materials, and outputs. Initially established within the funding of an NHMRC Partnership Project seeking to improve uptake of testing and treatment of blood-borne viruses and sexually transmissible infections among Aboriginal Australians, the panel has since been funded by UNSW to more broadly support community involvement in research. Panel coordinators (MW, KB, SG) work with community organisations and through other networks to publicise the panel. Community members self-nominate to the panel. The coordinators conduct an intake discussion with community members (typically over the telephone) and record their experiences that would be relevant to research projects, such as experience of injecting drug use, incarceration, sex work, and diagnosis of hepatitis C. Indigenous Australians are overrepresented in many of the population groups that are targeted by the research at UNSW. As a result, we have taken specific steps to engage a significant number of members who identify as Indigenous and with the lived experiences identified above which may be stigmatised within their own communities as well as within mainstream societies [8].

The panel was established at the end of 2016. With the support of UNSW funding since 2017, we have been able to diversify into others, including the establishment of a separate panel of people with lived experience of disability. We have recruited 147 members to the panel, of whom 59 identify as Aboriginal and/or Torres Strait Islanders. We continue to recruit the panel to ensure that we can meet researchers’ needs as some panel members may not be contactable for some periods (for example, due to mobility, lack of access to phone or internet data, and incarceration).

Researchers who wish to engage with the panel first discuss their requirements with the coordinators, who then select panel members with experiences that match the project. Coordinators conduct the consultation with panel members, as they have established a rapport with the members and this process maintains panel member anonymity and confidentiality. Following consultation, panel coordinators prepare a de-identified, consolidated report of responses for the researchers. Panel members are paid $40 for their expertise and time at each consultation. This amount is provided by the respective project being considered by the panel. Most researchers who have asked for input from the panel are from UNSW. However, we have begun to advertise the panel to researchers from other institutions.

Although this panel goes some way towards community involvement and consultation in the research process, it does not take the place of other aspects of community governance and ownership, especially as informed by principles of research with Indigenous peoples [9]. This panel is designed to canvas feedback from people with experiences that reflect the experiences of people who are likely to be research participants. Researchers are expected to seek other forms of community consultation and governance as appropriate to their research.

This model of community involvement addressed some of the structural barriers inherent in other models such as those built on an expectation of sustained involvement or face-to-face meetings over a long period that may be unattractive or exclude community members with least resources. We work in flexible ways that meet the needs and options of panel members. For example, we contact panel members via email, telephone, or post depending on their preference. We work with panel members with limited literacy and other issues to ensure that they can participate. This might mean, for example, reading documents to panel members, providing easy to read versions of documents and in appropriate font size. We welcome members back to the panel if they have not been able to participate as a result of mobility, unstable housing or incarceration. We have undertaken a number of consultations at a Sydney service for particularly marginalised people. Using these strategies, we want to ensure the inclusion of the voices and opinions of those who may otherwise be designated as “hard to reach”.

Panel members have been asked to provide comment on various aspects of research. This includes, for example, data collection instruments (surveys, interview schedules), participant information and consent forms, on the design of studies, analysis, and presentation of findings, and for projects which focus on Indigenous people, comments on aspects of the cultural appropriateness of the research question and approach.

A number of projects referred to the panel have had hepatitis C as a focus. Some panel members who are at risk of hepatitis C (via injecting drug use) have revealed during consultations that they are unaware of testing and treatment options. Panel coordinators have provided information about hepatitis C and suggested ways in which these members could engage with care. One of these panel members reported in a subsequent consultation that he had undergone testing for the first time and had also facilitated testing for his partner and child.

One of the guiding principles of the work of the panel is that the members experience this as a positive and respectful consultation. The panel coordinators are very experienced in community engagement and have their own experience with some of these issues in their lives or those of their families. Two of the coordinators identify as Aboriginal (MW and KB) and the third coordinator lives with a disability (SG). The panel members have expressed their pleasure at being involved in research with one remarking “no one has asked my opinion before”.

There are other aspects of the panel operations that we would like to progress. At the moment, we are not able to provide panel members with feedback on how their input has had an impact on the research. We would like to undertake a formal evaluation to ask researchers how the panel’s feedback has influenced their approach to this project and in general. Although we note previous lessons from the literature on the complexity of understanding the impact of consumer participation in research related to, for example, the diverse range of ways in which community involvement has been incorporated into research [10]. We would like to be able to support those panel members who can and who are interested to participate in other forums. However, not all panel members are interested in or available for such additional opportunities and are not willing to participate in activities that will reveal their identity.

## Conclusion

The literature about how to best “do” community involvement is still emerging and there are many possible ways in which to involve community in research. The UNSW Community Reference Panel provides a means to engage people all over the country in ways designed to meet their needs. We hope to contribute one more way to bring the voices and perspectives of people who are generally excluded from the research and decision-making structures that affect their lives, including people who inject drugs, to influence the questions that are asked in research, how research gets done, and to what purpose research findings are put.

## Data Availability

Not applicable
